# Transmission of Non-B HIV Subtypes in the United Kingdom Is Increasingly Driven by Large Non-Heterosexual Transmission Clusters

**DOI:** 10.1093/infdis/jiv758

**Published:** 2015-12-23

**Authors:** Manon Ragonnet-Cronin, Samantha J. Lycett, Emma B. Hodcroft, Stéphane Hué, Esther Fearnhill, Alison E. Brown, Valerie Delpech, David Dunn, Andrew J. Leigh Brown

**Affiliations:** 1 Institute for Evolutionary Biology; 2 Roslin Institute, University of Edinburgh, Edinburgh; 3 London School of Hygiene and Tropical Medicine; 4 MRC Clinical Trials Unit at University College London; 5 Public Health England, London, United Kingdom

**Keywords:** HIV, clusters, phylogenetics, subtypes, MSM, PWID, heterosexual, crossover, phylogeny, epidemiology

## Abstract

***Background.*** The United Kingdom human immunodeficiency virus (HIV) epidemic was historically dominated by HIV subtype B transmission among men who have sex with men (MSM). Now 50% of diagnoses and prevalent infections are among heterosexual individuals and mainly involve non-B subtypes. Between 2002 and 2010, the prevalence of non-B diagnoses among MSM increased from 5.4% to 17%, and this study focused on the drivers of this change.

***Methods.*** Growth between 2007 and 2009 in transmission clusters among 14 000 subtype A1, C, D, and G sequences from the United Kingdom HIV Drug Resistance Database was analysed by risk group.

***Results.*** Of 1148 clusters containing at least 2 sequences in 2007, >75% were pairs and >90% were heterosexual. Most clusters (71.4%) did not grow during the study period. Growth was significantly lower for small clusters and higher for clusters of ≥7 sequences, with the highest growth observed for clusters comprising sequences from MSM and people who inject drugs (PWID). Risk group (*P* < .0001), cluster size (*P* < .0001), and subtype (*P* < .01) were predictive of growth in a generalized linear model.

***Discussion.*** Despite the increase in non-B subtypes associated with heterosexual transmission, MSM and PWID are at risk for non-B infections. Crossover of subtype C from heterosexuals to MSM has led to the expansion of this subtype within the United Kingdom.

Of the multiple HIV subtypes and recombinant forms, subtype C predominates globally [1] but in the United Kingdom from the mid-1980s to around 1995, the epidemic was dominated by subtype B among men who have sex with men (MSM) [2]. Subtype B remains individually the most prevalent subtype in Europe (>80% of infections) [3] and in the United Kingdom (approximately 40% of diagnoses) [4]. However, non-B subtypes increased in prevalence in the United Kingdom, from <25% of diagnoses in the early 1990s [5] to 60% in 2010 [4]. Rises have been seen in other European countries [6–8], although in the United States only 3% of samples subtyped between 2004 and 2010 were non-B [9]. The increase in the United Kingdom and other European countries corresponds to growth in new HIV diagnoses among heterosexuals born abroad: however, after 2005, new diagnoses among heterosexuals born outside the United Kingdom decreased from 4426 to 2688 in 2013 [10].

By 2010, heterosexually acquired infections represented around half of United Kingdom diagnoses [10], of which 86% were non-B [4]. Subtype is frequently used as a proxy for transmission route in phylogenetic analyses of HIV [11, 12], although the prevalence of non-B subtype infections among United Kingdom–born MSM rose from 5.4% in 2007 [13] to 17% in 2010 [4].

Previous phylogenetic studies of subtype A and C sequences revealed United Kingdom–specific clusters [11], including among MSM [14], indicating some local transmission. Aggarwal et al investigated medical records and laboratory diagnostic findings for a small cohort to determine whether patients had been infected before or after arrival into the United Kingdom [15], but such an intensive approach is infeasible at the national level. Rice et al explored the origin of infections among heterosexuals born abroad, based on a detailed analysis of CD4^+^ T-cell count decline in combination with their date of entry in the country [16]. These authors estimated that 73% of HIV-positive heterosexuals born abroad with infection diagnosed in 2002 were infected outside the United Kingdom but that, by 2011, >50% were acquiring HIV within the United Kingdom [16]. This analysis was performed at the national level; however, it did not discriminate the subepidemics associated with different subtypes, and neither Aggarwal et al [15] nor Rice et al [16] could distinguish infections acquired through travel following domicile in the United Kingdom.

To analyze the recent transmission dynamics among non-B HIV subtypes in the United Kingdom, we used an automated phylogenetic clustering method [17]. We applied this approach to data from the United Kingdom HIV Drug Resistance Database (UKHIVRDB), which contains viral sequences from close to 50% of individuals in the United Kingdom with diagnosed HIV infection [4]. We quantified the transmission dynamics of non-B subtypes across risk groups in the United Kingdom.

## METHODS

For a description of the data set, see the Supplementary Table—Data.

### HIV Cluster Dynamics

#### Cluster Picking

We used the Cluster Picker and Cluster Matcher [17] to analyze transmission dynamics. Phylogenetic trees were built in RaxML [18] for each subtype separately against a background of global sequences from the Los Alamos National Laboratory HIV database (available at: http://www.hiv.lanl.gov). All A1, C, D, and G *pol* sequences (HXB2 nucleotide positions 2253–3459) with a minimum length of 500 base pairs, excluding those from the United Kingdom, were downloaded from the Los Alamos National Laboratory HIV database (>11 000 subtype C sequences and >4000 sequences each of subtypes A, D, and G). To limit the size of the trees, Viroblast [19] was used to select the 10 global background sequences closest to each UKHIVRDB sequence. In all 4 trees, clusters were picked if they contained at least 2 sequences, bootstrap support was >90%, and maximum pairwise genetic distance was ≤4.5%. A genetic distance threshold of 4.5% has previously been determined to best delineate epidemiologically relevant clusters in the United Kingdom [12]. We only analyzed clusters in which at least 80% of the sequences were from the United Kingdom, meaning that, for clusters of ≤4, all sequences had to be from the United Kingdom.

#### Cluster Dynamics

Clusters were sorted into risk groups based on self-identified risk group information associated with each sequence in the cluster. For clusters that contained at least 1 United Kingdom sequence collected up to December 2007, we counted the number of United Kingdom sequences in the cluster collected prior to December 2007 (hereafter, “old sequences”) and the number of United Kingdom sequences added to the cluster after 2007 (hereafter, “new sequences”). We calculated cluster growth as the number of new sequences divided by the number of old sequences, expressed as a percentage increment based on initial cluster size [20], and tested statistical significance using the Kruskal–Wallis test.

#### Simulations

We simulated phylogenies consistent with a simple epidemiological model in which all individuals in the population are equally likely to transmit, using custom R scripts according to the method described by Alizon et al [21]. The distribution of bootstrap support values and branch lengths were obtained from the full trees (excluding global background sequences), after which sequences collected after 2007 were stripped. New infections were then simulated as branching events in the tree. Given the incomplete coverage in the UKHIVRDB, the probability of any branch being selected was proportional to its length, to reflect the increased probability of missing HIV-positive individuals (regardless of whether infection was diagnosed) occurring along long branches. The branch length and bootstrap support for the new bifurcation were drawn at random from the branch length and bootstrap distributions of the full tree containing all sequences up to the end of 2009. Sequences were simulated along the resulting phylogenies under a generalized time-reversible substitution model with nucleotide frequencies from the original data by using SeqGen [22], ensuring that the mean genetic distance among simulated sequences was equivalent to that of the true sequences. This was repeated 1000 times for each tree. Clusters (≥90% bootstrap and ≤4.5% genetic distance) were picked in the simulated trees, and cluster growth was calculated as in the original trees, to generate expected cluster growth distributions for each subtype.

#### Generalized Linear Model (GLM)

We constructed a GLM expressing the number of new sequences as a variable dependent on the number of old sequences, risk group, and subtype. We first tested the distribution of the dependent variable against Poisson, negative binomial, and gamma distributions to verify that a GLM would be appropriate. The distribution of the number of new sequences was adequately fitted by a negative binomial distribution (*P* = .1, by the χ^2^ test), with no other model offering a better fit, and so we used the negative binomial GLM available in R.

All statistical analyses were conducted in R [23].

## RESULTS

We investigated the dynamics of transmission clusters of non-B subtype HIV sequences between January 2007 and December 2009 to determine the drivers of new diagnoses during this period. After exclusion of duplicates, the 2010 UKHIVRDB subtype A1 data set contained 2083 sequences, of which 630 were collected after December 2006, and the subtype C data set contained 10 830 sequences, including 4852 collected after 2006 (Supplementary Text—Data). Of 815 subtype D sequences, 279 were collected after 2006; and of 965 subtype G sequences, 472 were collected after 2006. Thus, while the UKHIVRDB subtype A1 and D data sets grew by approximately 50% during the study period, the subtype C and G data sets grew by >80%. To these were added >7000 non–United Kingdom sequences selected by Viroblast (see “Methods” section) to distinguish between United Kingdom and non–United Kingdom transmission.

### Cluster Composition

**Figure 1.**
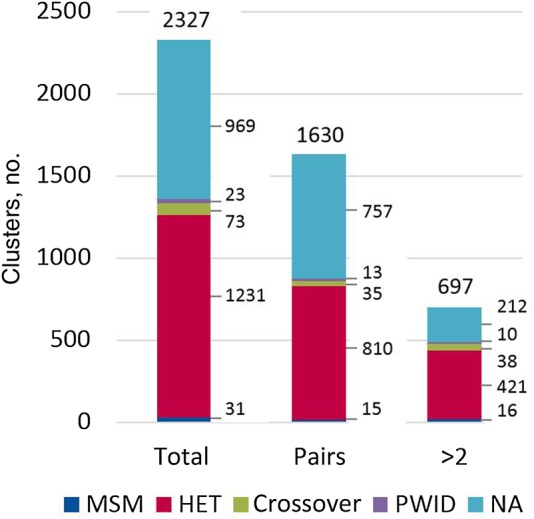
Risk group classification of clusters identified in 2009. A total of 2327 clusters (304 A1, 1785 C, 113 D, and 125 G) contained at least 2 sequences: 1630 clusters comprised pairs of sequences, and 697 comprised >2 sequences. Abbreviations: HET, heterosexual; MSM, men who have sex with men; PWID, people who inject drugs; NA, not available.

A total of 2327 clusters (304 A1, 1827 C, 112 D, and 125 G) meeting the above criteria were identified in the four trees. One hundred one clusters were excluded because <80% of the sequences therein were from the United Kingdom, of which only 26 contained >1 United Kingdom sequence. A total of 5999 United Kingdom sequences (41%) clustered with at least one other United Kingdom sequence used in the phylogenetic analysis. Over half of linked sequences were linked to only 1 other (hereafter, “pairs”; 3260 of 5999 sequences and 1630 of 2327 clusters). Clusters that contained >50% sequences with no risk group (969 of 2327 [42%]) were not classified. From a density plot to examine the risk group composition of each remaining cluster (Supplementary Figure 1), 4 groups clearly emerged. The majority of clusters (1231 of 1358 [91%]) contained sequences only from heterosexuals, and 31 clusters (2.3%) comprised sequences only from MSM. Seventy-three clusters (5.4%) contained sequences from both MSM and heterosexuals (at least 10% sequences from each). Finally, 24 clusters (1.7%) comprised sequences from persons who inject drugs (PWID; Figure 1). Some PWID clusters contained sequences from MSM and/or heterosexuals, but all contained at least 25% sequences from PWID. The breakdown by cluster size and risk group for clusters containing >1 risk group (all crossover clusters and PWID clusters that comprised >1 risk group) is shown in Supplementary Figure 2. We conducted a sensitivity analysis to test the effect of cluster classification on our results (Supplementary Figure 3).

### Cluster Growth Depends on Initial Cluster Size

Of the 1148 clusters that existed (pairs or larger clusters) in January 2007, only a minority (328 of 1148 [28.6%]) grew during the study period (Table 1). This effect was more pronounced in subtypes A1, D, and G, where <20% of clusters changed, compared with 33.5% in subtype C (*P* < .0001, by the Fisher exact test; Table 1). Pairs were significantly less likely to grow than larger clusters (*P* < .0001). Among pairs, only 215 of 879 (24.5%) changed during the study period. Overall, of 6233 new non-B subtype sequences added to the database after January 2007, 1457 (23.4%) linked to sequences already in the database before that date. This proportion was higher among subtypes A1 (24.6%) and C (25.0%) than among subtypes D (11.1%) and G (12.7%; *P* < .0001).

**Table 1.** Proportion of Clusters Showing Growth Between 2007 and 2009

HIV Subtype, Cluster Size	All Clusters, Proportion (%)	Clusters Displaying Growth, Proportion (%)
A1, C, D, G		
Overall^a^	1148/1148 (100)	328/1148 (28.6)
2 sequences	879/1148 (76.6)	215/879 (24.5)
A1		
Overall^a^	190/190 (100)	37/190 (19.5)
2 sequences	147/190 (77.4)	24/147 (16.3)
C		
Overall^a^	792/792 (100)	265/792 (33.5)
2 sequences	614/792 (77.5)	175/614 (28.5)
D		
Overall^a^	87/87 (100)	12/87 (13.8)
2 sequences	63/87 (72.4)	8/63 (12.7)
G		
Overall^a^	79/79 (100)	14/79 (17.7)
2 sequences	55/79 (69.6)	8/55 (14.5)

Abbreviation: HIV, human immunodeficiency virus.

^a^ Defined as those containing at least 1 sequence collected before December 2006.

**Figure 2.**
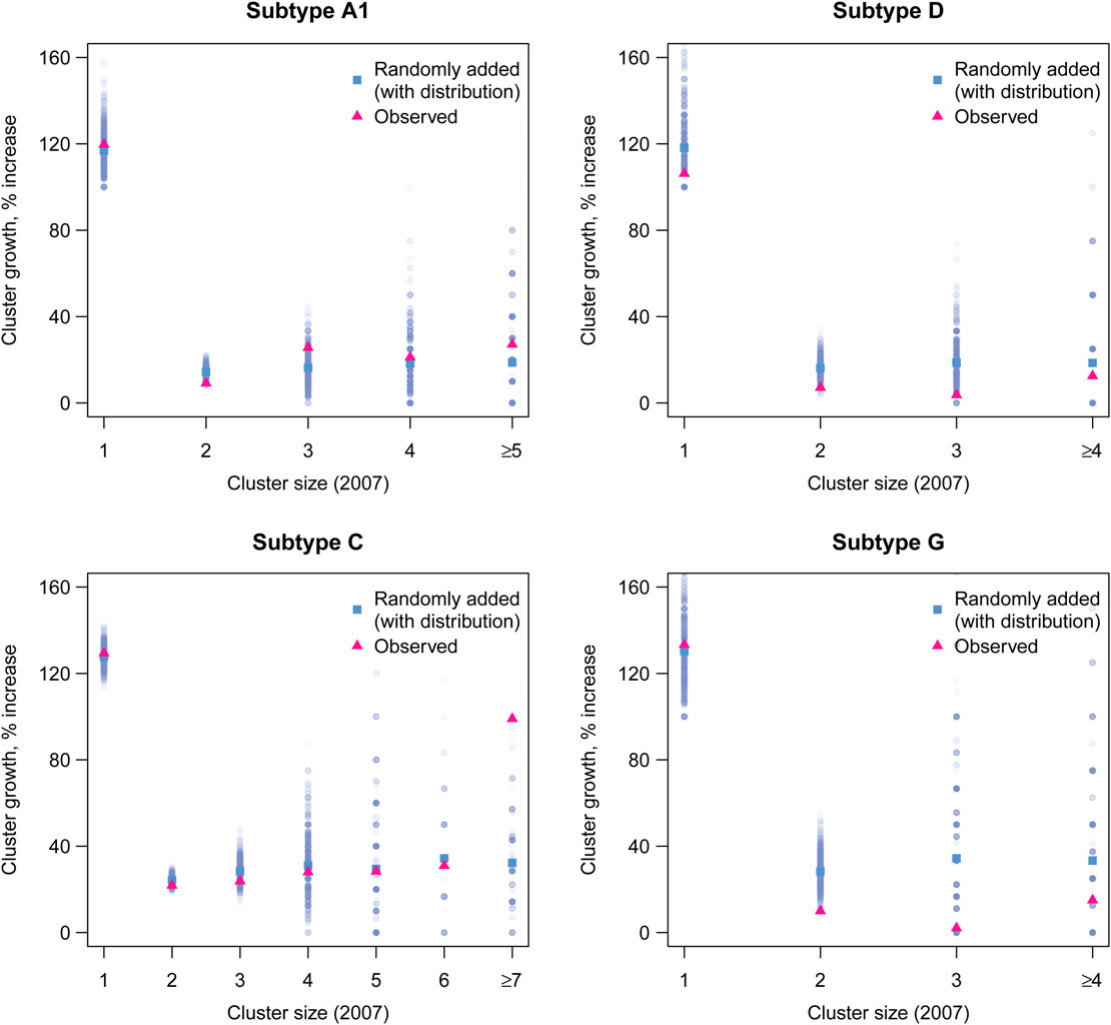
Cluster growth according to initial cluster size in 2007. For each cluster identified in 2009 containing at least 1 sequence collected before 2007, cluster growth was calculated as the number of new sequences divided by the number of old sequences (pink or black triangles). In parallel, we conducted simulations to generate expected cluster growth according to initial cluster size (blue or gray triangles). Data are from 1000 simulations, and mean values and distributions are shown. This figure is available in black and white in print and in color online.

For clusters containing at least 1 sequence collected prior to January 2007, we calculated growth as the number of sequences collected after this date divided by the number of sequences already clustered. In the United Kingdom data, we observed a significantly different degree of growth between clusters of different starting sizes (*P* < .0001, by the Kruskal–Wallis test; 4 degrees of freedom). We observed a trend of cluster growth increasing with cluster size in 2007 for subtypes A1 and C (Figure 2), which was significant only for subtype C (r = 0.95 and *P* < .05). Single sequences collected prior to 2007 that formed clusters after 2007 (total, 670 sequences) were not included.

We adopted a simulation-based approach to test whether observed cluster growth differed significantly from that expected if sequences were added to trees at random. If all individuals in a population are equally likely to transmit, the probability of a new infection linking to any specific infection from the original population is equal to 1 over the total size of the population. Although the entire population is not sampled, the full genetic diversity of the population is captured by the tree, and a new infection unlinked to those previously sampled will fall on a branch distant to the tree's terminal nodes. The longer the length of a single branch, the more likely it is that individuals have been missed along that branch and that a newly identified infection could occur along that branch. The probability of a new infection being linked to any given cluster is proportional to the size of that cluster.

Sequences collected after January 2007 were stripped from the tree and added back, with the probability of attachment based on the length of each branch and on the genetic distance and bootstrap distributions from the original tree (see Methods). In each simulated tree, clusters were picked as described previously, and the average cluster growth was calculated for clusters of each starting size and each risk group.

In the simulated data, average expected growth values were normally distributed (Figure 2). For clusters of each size, we evaluated whether the observed value of cluster growth fell within the 95% quantile estimates of the simulations (Table 2). For subtypes A1, D, and G, growth of pairs was below the 2.5% quantile (*P* < .05) of growth in the simulations. In 2007, there was a small number (8 of 792) of larger clusters (containing ≥7 sequences) in subtype C and none in the other subtypes. For this group, growth was higher than the 97.5% quantile (*P* < .05) of simulated clusters. All other values for cluster growth fell within the 95% quantiles of the simulations.

**Table 2.** Mean Observed Cluster Growth and Expected Growth by Cluster Size in 2007

HIV Subtype	Initial Cluster Size (2007)	Clusters, No.	Observed Growth	Expected Growth (2.5% Quantile)	Expected Growth (97.5% Quantile)
A1	2	147	0.094	0.097	0.196
C	7+	8	0.990	0.000	0.857
D	2	63	0.071	0.075	0.256
G	2	55	0.100	0.145	0.439

Clusters are only shown if their observed growth did not fall within the 95% expected growth quantiles (*P* < .05). Subtype C clusters with ≥7 sequences exceeded their expected growth, while subtype A, D, and G clusters with 2 sequences grew less than expected. Observed growth was calculated as the average growth for all clusters of the same initial cluster size in 2007. Expected growth for clusters of each initial size was estimated on the basis of 1000 simulations, and the 2.5% and 97.5% quantiles of each distribution are shown.

Abbreviation: HIV, human immunodeficiency virus.

### Cluster Growth Is Higher For Non-Heterosexual Risk Groups

**Figure 3.**
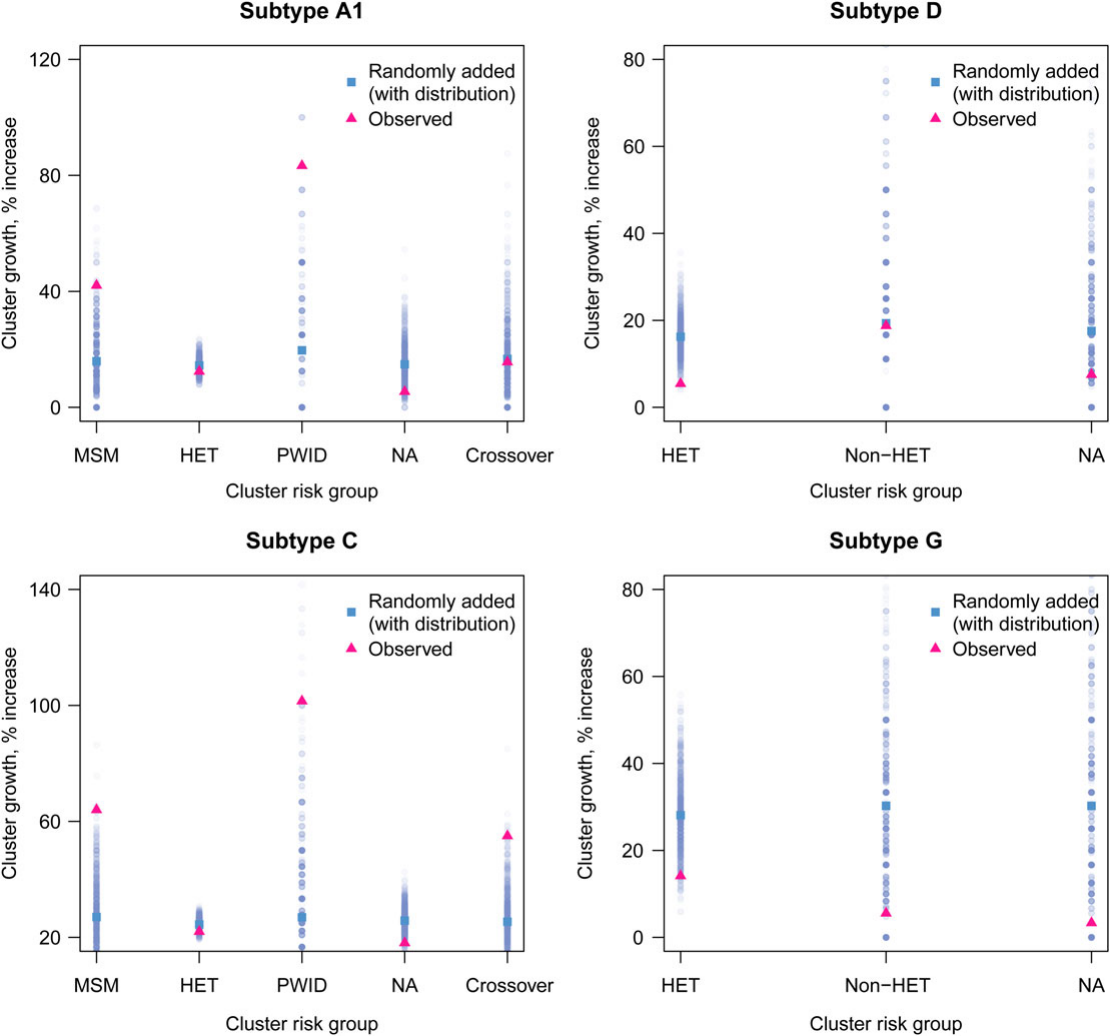
Cluster growth according to risk group. Observed cluster growth is shown as a pink or black triangle, and mean expected growth and distributions from 1000 simulations are shown in blue or gray. For subtypes D and G, because numbers of non-heterosexual (non-HET) clusters were small, these were amalgamated for analysis. Abbreviations: MSM, men who have sex with men; NA, not available; PWID, people who inject drugs. This figure is available in black and white in print and in color online.

Analysis by risk group was performed on clusters containing at least 2 sequences in 2007 (n = 1148). Growth differed significantly between risk groups when all subtypes were analyzed together (*P* < .005, by the Kruskal–Wallis test; 4 degrees of freedom). Although MSM clusters (mean growth, 54%), PWID clusters (mean growth, 64%), and crossover clusters (mean growth, 44%) grew significantly more than heterosexual clusters (mean growth, 18%), this was also observed in the simulated data (*P* < .0001; 4 degrees of freedom), owing to the differing initial cluster size between risk groups (*P* < .0001; 4 degrees of freedom). For subtypes A and C, cluster growth was higher in the observed data than in the simulations for PWID, MSM, and crossover clusters (Table 3 and Figure 3), indicating that cluster size was not the driver.

**Table 3.** Mean Observed Cluster Growth and Expected Growth by Risk Group

HIV Subtype	Risk Group	Clusters, No.^a^	Observed Growth	Expected Growth (2.5% Quantile)	Expected Growth (97.5% Quantile)
A1	MSM^b^	9	0.421	0	0.417
A1	PWID^b^	2	0.833	0	0.750
C	MSM^b^	10	0.640	0.050	0.550
C	PWID^b^	5	1.015	0	0.875
C	Crossover^b^	36	0.550	0.073	0.500
D	NA	31	0.075	0.358	1
G	Non-HET	6	0.056	0.130	0.462

Observed growth was calculated as the average growth for all clusters of the same risk group. Expected growth for clusters of each risk group was estimated on the basis of 1000 simulations, and the 2.5% and 97.5% quantiles of each distribution are shown.

Abbreviations: HIV, human immunodeficiency virus; MSM, men who have sex with men; NA, not available; non-HET, non-heterosexual; PWID, people who inject drugs.

^a^ Defined as clusters containing at least 2 sequences collected before January 2007. Clusters are only shown if their observed growth did not fall within the 95% expected growth quantiles (*P* < .05).

^b^ Groups with clusters that grew more than expected.

### Cluster Size and Risk Group Act Independently on Cluster Growth

To determine whether the effect of cluster size remained significant when risk group was accounted for, we constructed a GLM expressing the number of new sequences as a variable dependent on the number of old sequences, risk group, and subtype. Risk group and number of old sequences were added to the model sequentially, with risk group highly predictive (*P* < .0001) and the addition of cluster size significantly improving the fit of the model (*P* < .0001, by the analysis of variance likelihood ratio test). The difference in effect between subtypes was highly significant (*P* < .0001). There was no significant interaction between risk group and cluster size (*P* = .108). In terms of effect, the model explained 24.75% of the variance in growth: risk group accounted for 12.31%, initial cluster size for 9.32%, and subtype for 3.12%. Cluster growth was higher for crossover clusters (*P* < .0001), PWID clusters (*P* < .05), and MSM clusters (*P* < .01); higher for subtype C clusters (*P* < .01); and increased with initial cluster size (*P* < .0001).

### HIV Subtype C Is Increasingly Acquired in the United Kingdom, While Subtypes D and G Are Imported

The ratio of clustering to nonclustering subtype C sequences increased over time, indicating a rise in the proportion of local subtype C transmissions, while this ratio decreased for subtypes D and G (Supplementary Figure 4). For all clusters in 2009 containing at least 1 sequence in 2007, we counted the number of new sequences clustering in crossover and MSM clusters (Table 4). When we looked at the proportion of new diagnoses linking to crossover and MSM clusters (2%–3% of new diagnoses) as opposed to HET clusters, proportions were not different across subtypes (*P* = .38, by the Fisher exact test), although numbers were small for subtypes D and G, with only 90 new diagnoses linking to subtype D and G clusters.

**Table 4.** Number of Sequences Added to Clusters Between 2007 and 2009, According to Risk Group and to Cluster Size in 2007

HIV Subtype, Initial Cluster Size	Crossover	HET	PWID	MSM	NA	Total
A1						
Overall	7/630 (1.11)	76/630 (12.1)	7/630 (1.11)	14/630 (2.22)	51/630 (8.1)	155/630 (24.6)
1 sequence	2	41	2	2	44	91
≥2 sequences	5	35	5	12	7	64
C						
Overall	81/4852 (1.67)	620/4852 (12.78)	38/4852 (0.78)	40/4852 (0.82)	432/4852 (8.9)	1211/4852 (24.96)
1 sequence	15	362	5	7	320	709
≥2 sequences	66	258	33	33	112	502
D						
Overall	2/279 (0.72)	14/279 (5.02)	1/279 (0.36)	1/279 (0.36)	13/279 (4.66)	31/279 (11.11)
1 sequence	0	8	1	1	7	17
≥2 sequences	2	6	0	0	6	14
G						
Overall	9/472 (1.91)	30/472 (6.36)	3/472 (0.64)	0/472 (0)	18/472 (3.81)	60/472 (12.71)
1 sequence	9	14	2	0	15	40
≥2 sequences	0	16	1	0	3	20
All subtypes						
Overall	99/6233 (1.59)	740/6233 (11.87)	49/6233 (0.79)	55/6233 (0.88)	514/6233 (8.25)	1457/6233 (23.38)
1 sequence	26	425	10	10	386	857
≥2 sequences	73	315	39	45	128	600

Data are no. of sequences added to all clusters of the risk group per cluster size of interest or no. of sequences added to all clusters of the risk group per cluster size of interest/total no. of new sequences for each subtype (%).

Abbreviations: HET, heterosexual; HIV, human immunodeficiency virus; MSM, men who have sex with men; NA, not available; PWID, people who inject drugs.

## DISCUSSION

We have developed a novel phylogeny-based approach to generate null patterns of cluster growth with which to compare observed growth. This method has the benefit of not requiring all the infections to be sampled, because the full genetic diversity of the population is still captured by the phylogenetic tree. At least 25% of non-B subtype infections diagnosed between 2007 and 2009 were linked to previously diagnosed infections and are likely to have been acquired within the United Kingdom. Among preexisting clusters, we identified that cluster size and risk group each independently predicted higher cluster growth. We conclude that crossover to non-heterosexual risk groups has resulted in increased United Kingdom–based transmission of subtype C in particular, as has been observed in France [24]. However, the majority of clusters did not grow, and there were important differences between the subtypes analyzed.

Subtypes C, A1, D, and G are the most common subtypes in the United Kingdom, after subtype B, and are found predominantly among heterosexuals [11]. Subtype C infections are most common in South Africa, subtype A and D infections are most common in East Africa, and subtype G infection is most common in West Africa [1]. Subtype A has entered the United Kingdom in parallel from Eastern Europe, where it is transmitted among PWID [25], and this may account for the slightly higher proportion of new subtype A sequences linking to PWID clusters, compared with other subtypes (1.11% vs 0.78% for subtype C). Around half of diagnoses in the United Kingdom every year are now non-B subtypes, with subtype C alone accounting for one third [4]. We found the overwhelming majority of clusters to comprise sequences from heterosexuals, as expected. However, clusters classified as comprising sequences from MSM or PWID were significantly more likely to link to new infections. Crossover clusters containing sequences from both heterosexuals and MSM were also more likely to grow. For subtype C, the number of newly clustering sequences in crossover clusters greatly exceeded the expected number, based on the simulations and the clustering ratio increased, while the less abundant subtypes D and G remained dominated by imports (Supplementary Figure 4). Crossover of subtype B from MSM into heterosexuals has previously been reported in the United Kingdom [26], with Black-African men clustering exclusively with MSM more likely to self-identify as heterosexuals, compared with other ethnicities. Such mixing could be responsible for the spread of non-B subtypes among MSM. In contrast, the epidemic growth of non-B subtypes among heterosexuals in the United Kingdom appears limited, as previously suggested [16].

It is noteworthy that most preexisting clusters (>70%) did not link to any new infections. Subtype A1, D, and G pairs grew less than expected, with 85% not changing at all. The observation that larger clusters act as a driving force for epidemic growth is consistent with the hypothesis of preferential attachment [27], whereby already highly connected individuals tend to make proportionally more contacts over time. The United Kingdom subtype B epidemic among MSM has previously been suggested to be driven through a process of preferential attachment [27], but because the MSM and heterosexual epidemics have different epidemic structure [11, 12], we compared cluster growth to a null model in which every person was equally likely to transmit. The GLM analysis showed that cluster size remained significant in the model even when risk group was taken into account. One explanation for this phenomenon could be that larger clusters comprise sequences from acutely infected individuals who drive new infections [28, 29].

Although the relative frequencies of A1, C, D, and G in the United Kingdom reflect their respective global prevalence [1], we observed significant differences in their patterns of growth. Thus, the United Kingdom subtype G epidemic grew as much as the subtype C epidemic (ie, >80% during the study period), but most did not link to previous United Kingdom infections. The ratio of clustering to nonclustering sequences for D and G decreased between 2007 and 2009; we conclude that D and G were not being transmitted as much as C within the United Kingdom. Meanwhile, several large clusters of subtype C sequences from non-heterosexuals displayed cluster growth more similar to that of subtype B [12], and numerous crossover events were observed. We conclude that accelerated spread of non-B subtypes has followed transmission into non-heterosexuals.

Our figure of 25% is, for several reasons, an underestimate of the proportion of non-B strains acquired within the United Kingdom. We will miss some infections acquired within the United Kingdom because of late diagnosis and because of the choice of clustering threshold, which may exclude some links erroneously [30]. Heterosexuals are known to present later during infection than MSM [10], and immigrant groups may be particularly slow to engage with healthcare providers. Nevertheless, the representation of the UKHIVRDB is high, and over time it will accumulate even late-presenting patients, so linkages will increase. In addition, while there is an element of arbitrariness in any selection of specific dates, choosing a cutoff of January 2007 equalized the number of sequences on either side in the 2010 release of the UKHIVRDB. As time-resolved trees of the United Kingdom non-B epidemic previously showed that sequences were added to clusters linearly with time [11] the choice of cutoff is unlikely to influence results. The choice of bootstrap and genetic distance cutoffs is subjective, but the thresholds used here have been shown to appropriately delineate epidemiologically relevant clusters in the United Kingdom [12]. The same clusters are identified at a range of thresholds [17], and so our findings are unlikely to be affected by our choice of threshold. Finally, formal methods linking phylogenetic reconstructions and epidemiological models remain difficult to apply to large data sets. However, our use of simulations as a null model to compare our data to has provided a direct test and demonstrated that growth rates of clusters containing sequences from non-heterosexuals diverge dramatically from a null model in which all individuals in the population are equally likely to transmit. In previous analyses of cluster dynamics over time, a formal comparison of observed cluster growth to the growth expected given the change in the number of sequences was not included [20, 28]. Brenner et al analyzed time-resolved trees, which might be expected to improve estimation of the timing of transmission events, but the method used here, which is based on the dates of diagnosis, is applicable to much larger data sets [28].

Overall, we found that the majority of clusters within the United Kingdom non-B epidemic did not grow between 2007 and 2009 and those that did were more likely to already be larger and to contain sequences from MSM or PWID. Subtype C infections are increasingly being acquired within the United Kingdom, while subtypes D and G infections remain dominated by importation. We conclude that crossover into non-heterosexual risk groups by 2010 led to rapid expansion of non-B subtypes within the United Kingdom, a trend likely to have continued. This study underlines the importance of continuing efforts to prevent HIV transmission among all risk groups despite the United Kingdom epidemic changing from predominately involving MSM and subtype B to being more diverse in terms of risk group and subtypes.

## Supplementary Data

Supplementary materials are available at http://jid.oxfordjournals.org. Consisting of data provided by the author to benefit the reader, the posted materials are not copyedited and are the sole responsibility of the author, so questions or comments should be addressed to the author.

Supplementary Data
