# Supplementary material for: Transmission of Non-B HIV Subtypes in the United Kingdom Is Increasingly Driven by Large Non-Heterosexual Transmission Clusters
Source: J Infect Dis. 2015 Dec 23;213(9):1410–8. doi: 10.1093/infdis/jiv758 (PMC4813743; doi:10.1093/infdis/jiv758)
Supplement: Supplementary Data [file supp_213_9_1410__index.html]

Transmission of Non-B HIV Subtypes in the United Kingdom Is Increasingly Driven by Large Non-Heterosexual Transmission Clusters — Supplementary Data 

# Transmission of Non-B HIV Subtypes in the United Kingdom Is Increasingly Driven by Large Non-Heterosexual Transmission Clusters

## Supplementary Data

Supplementary Data

- Supplementary Data\_file1 - docx file
- Supplementary Data\_file2 - pdf file
