# Supplementary material for: Transmission of Non-B HIV Subtypes in the United Kingdom Is Increasingly Driven by Large Non-Heterosexual Transmission Clusters
Source: J Infect Dis. 2015 Dec 23;213(9):1410–8. doi: 10.1093/infdis/jiv758 (PMC4813743; doi:10.1093/infdis/jiv758)
Supplement: Supplementary Data [file supp_jiv758_jiv758supp_Data_file1.docx]

**UK HIV Drug Resistance Database**

**Steering Committee:** Celia Aitken (Gartnavel General Hospital, Glasgow); David Asboe, Anton Pozniak (Chelsea & Westminster Hospital, London); Patricia Cane (Public Health England, Porton Down); David Chadwick (South Tees Hospitals NHS Trust, Middlesbrough); Duncan Churchill (Brighton and Sussex University Hospitals NHS Trust); Duncan Clark (St Bartholomew's and The London NHS Trust); Simon Collins (HIV i-Base, London); Valerie Delpech (Centre for Infections, Public Health England); Samuel Douthwaite (Guy’s and St. Thomas’ NHS Foundation Trust, London); David Dunn, Esther Fearnhill, Kholoud Porter, Anna Tostevin, Ellen White (MRC Clinical Trials Unit at UCL, London); Christophe Fraser (Imperial College London); Anna Maria Geretti (Institute of Infection and Global Health, University of Liverpool); Antony Hale (Leeds Teaching Hospitals NHS Trust); Stéphane Hué (University College London); Steve Kaye (Imperial College, London); Paul Kellam (Wellcome Trust Sanger Institute & University College London Medical School); Linda Lazarus (Expert Advisory Group on AIDS Secretariat, Public Health England); Andrew Leigh-Brown (University of Edinburgh); Tamyo Mbisa (Virus Reference Department, Public Health England); Nicola Mackie (Imperial NHS Trust, London); Samuel Moses (King’s College Hospital, London); Chloe Orkin (St. Bartholomew's Hospital, London); Eleni Nastouli, Deenan Pillay, Andrew Phillips, Caroline Sabin (University College London Medical School. London); Erasmus Smit (Public Health England, Birmingham Heartlands Hospital); Kate Templeton (Royal Infirmary of Edinburgh); Peter Tilston (Manchester Royal Infirmary); Daniel Webster (Royal Free NHS Trust, London); Ian Williams (Mortimer Market Centre, London); Hongyi Zhang (Addenbrooke’s Hospital, Cambridge).

**Coordinating Centre:** MRC Clinical Trials Unit at UCL (Hannah Castro, David Dunn, Keith Fairbrother, Esther Fearnhill, Kholoud Porter, Anna Tostevin, Ellen White)

**Centres contributing data:** Clinical Microbiology and Public Health Laboratory, Addenbrooke’s Hospital, Cambridge (Jane Greatorex); Guy’s and St. Thomas’ NHS Foundation Trust, London (Siobhan O’Shea, Jane Mullen); PHE – Public Health Laboratory, Birmingham Heartlands Hospital, Birmingham (Erasmus Smit); PHE – Virus Reference Department, London (Tamyo Mbisa); Imperial College Health NHS Trust, London (Alison Cox); King's College Hospital, London (Richard Tandy); Medical Microbiology Laboratory, Leeds Teaching Hospitals NHS Trust (Tracy Fawcett); Specialist Virology Centre, Liverpool (Mark Hopkins, Lynn Ashton); Department of Clinical Virology, Manchester Royal Infirmary, Manchester (Peter Tilston); Department of Virology, Royal Free Hospital, London (Claire Booth, Ana Garcia-Diaz); Edinburgh Specialist Virology Centre, Royal Infirmary of Edinburgh (Jill Shepherd); Department of Infection & Tropical Medicine, Royal Victoria Infirmary, Newcastle (Matthias L Schmid, Brendan Payne); South Tees Hospitals NHS Trust, Middlesbrough (David Chadwick); Department of Virology, St Bartholomew’s and The London NHS Trust (Spiro Pereira, Jonathan Hubb); Molecular Diagnostic Unit, Imperial College, London (Steve Kaye); University College London Hospitals (Stuart Kirk); West of Scotland Specialist Virology Laboratory, Gartnavel, Glasgow (Rory Gunson, Amanda Bradley-Stewart, Celia Aitken).
