# Supplementary material for: Transmission of Non-B HIV Subtypes in the United Kingdom Is Increasingly Driven by Large Non-Heterosexual Transmission Clusters
Source: J Infect Dis. 2015 Dec 23;213(9):1410–8. doi: 10.1093/infdis/jiv758 (PMC4813743; doi:10.1093/infdis/jiv758)
Supplement: Supplementary Data [file supp_jiv758_jiv758supp_Data_file2.pdf]

Supplementary Figure 1: Density plots of risk group composition for each cluster

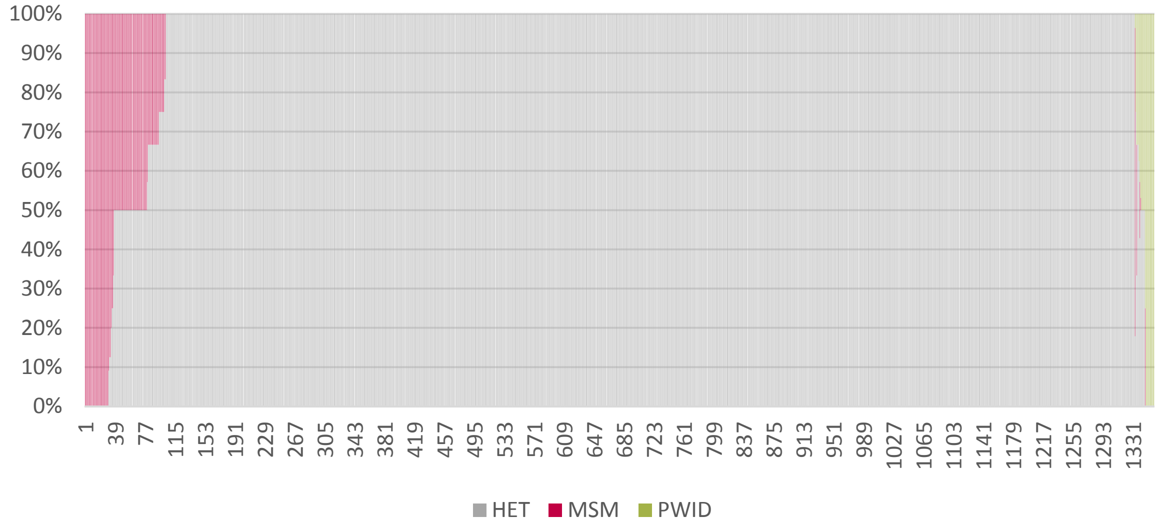

Each vertical line represents a separate cluster, and clusters are sorted by proportion of heterosexuals (HET), men who have sex with men (MSM) and people who inject drugs (PWID). Clusters which could not be classified (>50% sequences with no risk group) and sequences for which risk group was not available are not shown within cluster composition. Four groups emerged clearly: clusters which were fully heterosexual (1230/1358% of clusters), clusters which were fully MSM (31/1358,% of clusters), clusters which were a mix of heterosexuals and MSM (73/1358 % of clusters) and clusters which contained PWID (24/1358). Some PWID clusters contained MSM and heterosexuals but all contained at least 25% sequences from PWID.

Supplementary Figure 2: Breakdown by cluster size and risk group for clusters that contained more than one risk group

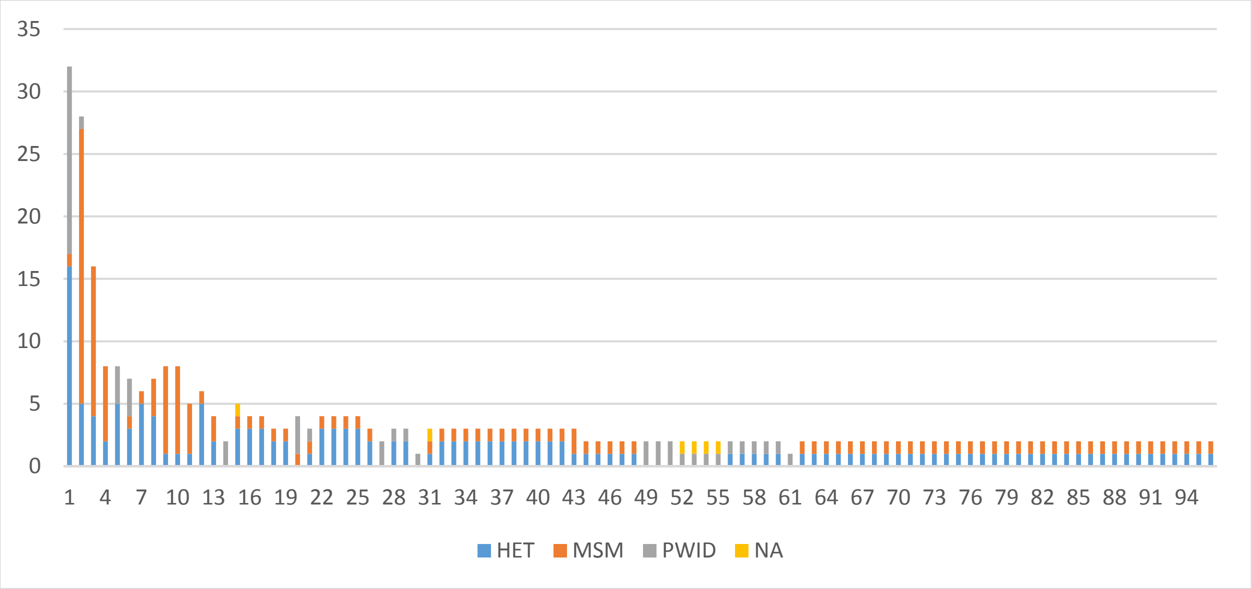

HET: heterosexual, MSM: men who have sex with men, PWID: people who inject drugs, NA: not available. The figure includes all 73 crossover clusters and 23/24 PWID clusters. Clusters are sorted by size.

Supplementary Figure 3: Growth rate according to risk group under three definitions

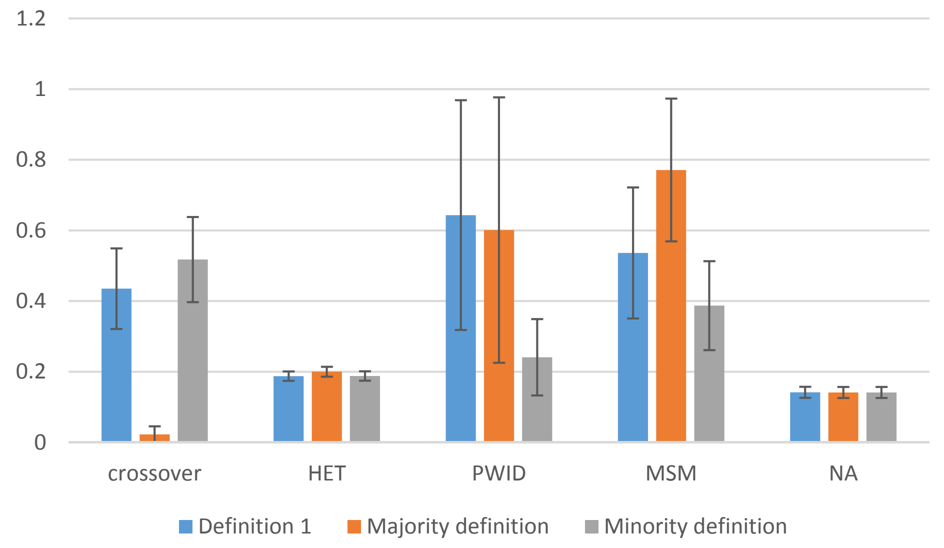

MSM: men who have sex with men, HET: heterosexual, PWID: people who inject drugs; NA not available.

Two risk group classification procedures (minority and majority definition) were tested in addition to the classification used in the paper. According to our majority risk group definition, the risk group of the cluster was that of the majority of the sequences in the cluster. If two risk groups each accounted for 50% of sequences, both risk groups were used and growth was divided proportionally between them (or attributed to the crossover risk group in the case of HET-MSM clusters). According to the minority cluster definition, the risk group of any sequence in the cluster entered the risk group classification and growth was divided proportionally between them (or attributed to the crossover risk group in the case of HET-MSM clusters). In both cases, clusters containing 50% or more sequences with unknown risk group were classified as NA.

Changing the rules of cluster risk group classification changed the risk group of only 11/1148 clusters for the minority definition and 43/1148 clusters for the majority definition (of which 29 were crossover clusters which became either HET or MSM). Differences in growth rates between risk groups were unchanged (as shown by the overlap in standard error bar) other than crossover growth rate dropping in the majority definition because most crossovers clusters were relabelled as either MSM or HET.

Supplementary Figure 4: Change in clustering ratio between 2007 and 2009

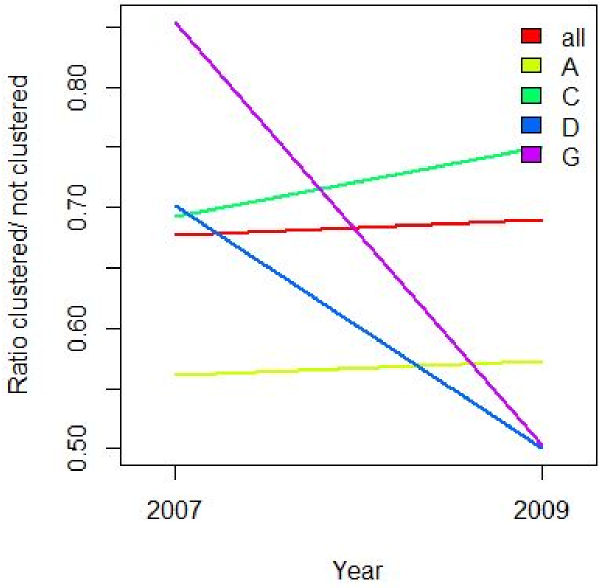

An increase in the ratio of clustering to non-clustering sequences in the database over time would indicate a rise in the proportion of local transmissions. When all subtypes were analysed together, this ratio did increase but the change was not significant (Cochran-Armitage test across years [1, 2], p=0.5). Broken down by subtype, the subtype C clustering ratio rose from 0.69 to 0.75 (p=0.01), indicating an increasing proportion of infections acquired within the UK over time. In contrast, the clustering ratio decreased for D (from 0.70 to 0.50; p<0.01) and G (from 0.85 to 0.50; p<0.0001), signifying that most new sequences were unlinked to those already in the UK and are more likely to be the result of migration. However, overall numbers were small, with only 279 subtype D and 472 subtype G diagnoses after 2006. The change was not significant for A1 (p=0.7).

Reference List

1. Armitage P. Tests for Linear Trends in Proportions and Frequencies. Biometrics **1955**;11:375-86.

2. Cochran WG. Some methods for strengthening the common X^2 tests. Biometrics **1954**;10:417-51.

Data

HIV pol sequences from the UK HIV Drug Resistance Database analysed for this study

Listed by subtype

B	C	A1	G	D	Other	Total
22057<br><br>(52.3%)	10872<br><br>25.3%	2083<br><br>(4.9%)	965<br><br>(2.2%)	815<br><br>(1.9%)	6210<br><br>(14.4%)	43002

The UK HIV Drug Resistance Database receives sequences of the pol region obtained for routine clinical surveillance and submitted by participating laboratories. Sequences were originally obtained at virological failure, then at the onset of therapy and most recently at diagnosis, according to the prevailing guidelines. Sequences are available for around 50% of the infected population and >80% of patients diagnosed since 2005. For non-B subtypes, 80% of sequences in the database are from samples collected after 2005. 43,002 partial HIV *pol* sequences were obtained from the UKHIVRDB (2010 download; sequences up to end of 2009). The majority of sequences (>90%) covered the entire protease gene and up to 900 bases of reverse transcriptase. Sequences generated using the Trugene® assay were missing the first 120 nucleotides (40 amino acids) of reverse transcriptase. Epidemiological data contributed by Public Health England included year of birth, gender and self-reported most likely route of infection (people who inject drugs (PWID), heterosexual sex (HET), MSM, mother to child, blood product, or unknown). Epidemiological data and sequences were linked using partial identifiers, then the data were fully anonymised and delinked before phylogenetic analysis. For patients with more than one sequence in the database the earliest sequence was used, usually obtained before the initiation of antiretroviral therapy. Subtypes were assigned using SCUEAL [1]. Of 43,002 sequences, 22,507 (52.3%) were classified as subtype B. Subtypes C (10872,

25.3%), A1 (2083, 4.9%), G (965, 2.2%) and D (815, 1.9%) were the next most common and are analysed here. The UKHIVRDB sequences have in the past been subtyped with REGA [2] and agreement between SCUEAL and REGA has been excellent for pure subtypes [1]. Sequences were stripped of 45 sites associated with drug resistance based on the 2011 International AIDS Society-USA drug resistance list [3]. Identical sequences were removed using ElimDupes . (http://www.hiv.lanl.gov/content/sequence/ELIMDUPES/elimdupes.html) as duplicate sequences are highly unlikely to come from different patients.

As submission of the entire sequence dataset to public databases would permit transmission network identification and thus risk breaching patient confidentiality, we have followed earlier practice [4] and submitted a random sample of 10% of each subtype to GenBank under accession numbers KU498303 - KU499411.

The following previously submitted sequences were also included in this study: Q462027-Q462034; Q462036; Q462040; Q462042; Q462044-Q462047; Q462049-Q462052; Q462054; Q462056-Q462060; Q462062-Q462065; Q462067; Q462070-Q462077; Q462079; Q462081-Q462091; Q462093-Q462098; Q462100-Q462105; Q462107; Q462109; Q462111-Q462113; Q462115; Q462116; Q462118-Q462129; Q462133; Q462134; Q462137; Q462139-Q462145; Q462147-Q462150; Q462152-Q462161; Q462163-Q462173; Q462175-Q462179; Q462182-Q462187; Q462189; Q462191; Q462192; Q462195-Q462200; Q462202; Q462208; Q462209; Q462212; Q462213; Q462216; Q462217; Q462219; Q462222; Q462223; Q462226-Q462231; Q462234; Q462235; Q462237-Q462245; Q462247; Q462248; Q462254; Q462258; Q462260-Q462262; Q462264-Q462267; Q462269; Q462270-Q462278; Q462280; Q462282; Q462283; Q462285; Q462287; Q462289-Q462292; Q462294; Q462295; Q462299; Q462300-Q462302; Q462305; Q462306; Q462308-Q462316; Q462318-Q462322; Q462324-Q462335; Q462337-Q462344; Q462346-Q462353; Q462355-Q462359; Q462363-Q462367; Q462369; Q462371-Q462377; Q462380-Q462383; Q462385; Q462387; Q462392-Q462401; Q462403; Q462404; Q462406-

Q462408; Q462411-Q462419; Q462423-Q462426; Q462428; Q462433; Q462434; Q462437; Q462441; Q462443-Q462448; Q462450-Q462452; Q462454; Q462455; Q462457; Q462458; Q462460; Q462461; Q462463-Q462468; Q462471; Q462473-Q462477; Q462481-Q462489; Q462491; Q462492; Q462496-Q462511; Q462514-Q462516; Q462518; Q462521-Q462524; Q462526; Q462527; Q462529-Q462532.

Ethical approval was given by the London Multicentre Research Ethics Committee (MREC/01/2/10; 5 April 2001). Data held in the UKHIVRDB can be accessed for collaborative projects approved by the Steering Committee. The proposal form can be downloaded from www.hivrdb.org.

Reference List

1. Kosakovsky Pond SL, Posada D, Stawiski E, Chappey C, Poon AF, Hughes G, et al. An evolutionary model-based algorithm for accurate phylogenetic breakpoint mapping and subtype prediction in HIV-1. PLoS Comput Biol **2009**;5:e1000581.

2. de Oliveira T, Deforche K, Cassol S, Salminen M, Paraskevis D, Seebregts C, et al. An automated genotyping system for analysis of HIV-1 and other microbial sequences. Bioinformatics **2005**;21:3797-800.

3. Johnson VA, Calvez V, Gunthard HF, Paredes R, Pillay D, Shafer R, et al. 2011 update of the drug resistance mutations in HIV-1. Top Antivir Med **2011**;19:156-64.

4. Leigh Brown AJ, Lycett SJ, Weinert L, Hughes GJ, Fearnhill E, Dunn DT. Transmission network parameters estimated from HIV sequences for a nationwide epidemic. J Infect Dis **2011**;204:1463-9.
